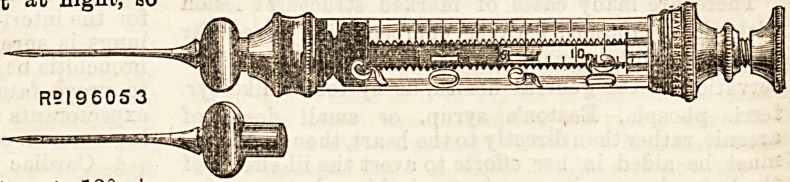# New Drugs, Appliances, and Things Medical

**Published:** 1892-12-31

**Authors:** 


### NEW DRUGS, APPLIANCES, AND THINGS MEDICAL.

### AN ANTISEPTIC HYPODERMIC SYRINGE.

The modern surgeon is aseptic in all his methods. Antiseptic treatment is regarded as the subterfuge of the uncleanly, and an anachronism in these days of super-heated steam and high temperature ovens. Aseptic methods have, indeed, up to the present been regarded as the exclusive monopoly of the surgeon. Messrs. Meyer and Meltzer have, however, given an opening to the physician for a truly aseptic method by the introduction to the profession of a new syringe for hypodermic injection. The old hypodermic syringe has from time to time proved a dangerous instrument in the hands of the careless. The instrument is a difficult one to keep absolutely clean, and if the needle, as occasionally happens, becomes blocked with coagulated blood, it offers serious, if not insurmountable, difficulties to the would-be cleaner; if such a needle be then subcutaneously inserted

Rº 19605

under the skin, blood poisoning and death may ensue. To provide against this contingency, Messrs. Meyer and Meltzer have advertised a new syringe, the needle of which may be

Rº 19605A

completely and absolutely cleaned and rendered entirely aseptic by sterilization in the naked flame. The syringe itself is like many others that have been offered to the pro-

Rº196053

fession, and if it has not all the modern improvements, it is at least a good one. Its advantages are so obvious, that it would be waste of time to enumerate them. We ourselves use one constantly in the wards of a large London hospital and consider it safer than any other form of syringe.

## Figures and Tables

**Figure f1:**
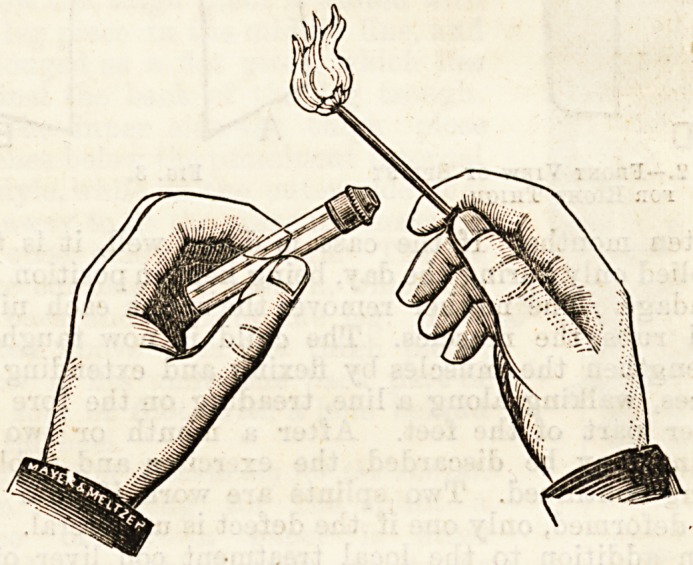


**Figure f2:**
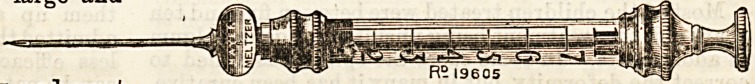


**Figure f3:**
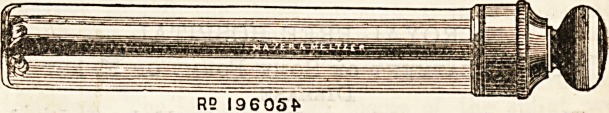


**Figure f4:**